# Family planning utilization among people living with HIV: a cross-sectional study at the Kumasi South Hospital, Ghana

**DOI:** 10.11604/pamj.2026.53.137.43954

**Published:** 2026-03-24

**Authors:** John Jude Kweku Annan, Boniface Mensah, Betty Roberta Norman, Rachel Agyeman Boateng, Anthony Enimil, Christiana Asiedu-Neizer, Martin Agyei, Tracey Afoley Laryea

**Affiliations:** 1 Department of Obstetrics and Gynecology, School of Medical Sciences, College of Health Sciences, Kwame Nkrumah University of Science and Technology, Kumasi, Ghana,; 2 Department of Internal Medicine, Kumasi South Hospital, Kumasi, Ghana,; 3 Department of Internal Medicine, School of Medical Sciences, College of Health Sciences, Kwame Nkrumah University of Science and Technology, Kumasi, Ghana,; 4 Department of Internal Medicine, The Police Hospital, Accra, Ghana,; 5 Department of Child Health, School of Medical Sciences, College of Health Sciences, Kwame Nkrumah University of Science and Technology, Kumasi, Ghana,; 6 Directorate of Internal Medicine, Komfo Anokye Teaching Hospital, Kumasi, Ghana

**Keywords:** Family planning utilisation, modern family planning, HIV infections, contraception, persons living with HIV

## Abstract

**Introduction:**

prevention of unintended pregnancies among Women Living with Human Immunodeficiency Virus (WLWHIV) through family planning is a highly cost-effective vertical transmission prevention strategy. This study evaluated the family planning utilization and fertility needs among adults Persons Living with HIV (PLWHIV) in a regional hospital in Ghana.

**Methods:**

this was a cross-sectional study conducted at the HIV clinic of Kumasi South Hospital, a regional hospital in Ghana. Study participants (n=602) were sampled and interviewed using questionnaires to determine the consistent use of contraception. A descriptive analysis was done to determine the rate of consistent contraceptive use. A multivariable analysis was used to assess factors affecting the consistent use of contraception among PLWHIV at a statistical significance of 0.05.

**Results:**

barrier contraception is the most well-known and widely used among study participants (>98%). Factors associated with increased odds of consistent use of contraception included marital status, HIV infection, duration of 4 or more years, and receipt of family planning assistance. However, having a seropositive sexual partner was associated with less consistent use of contraception.

**Conclusion:**

barrier contraception, the most popular method among PLHIV, is less effective in reducing unintended pregnancies. Efforts to meet the contraception needs of PLHIV should be focused on dual contraception (barrier and long-term reversible methods) to reduce HIV transmission, dual HIV infections, and unintended pregnancies.

## Introduction

Globally, an estimated 31.6-44.5 million people were living with HIV infection at the end of 2019. The greatest burden of HIV is in sub-Saharan Africa, where nearly 1 in every 25 adults lives with HIV, accounting for more than two-thirds of HIV infection worldwide [1]. In sub-Saharan Africa, women and girls accounted for 59% of all new HIV infections and almost 60% of the total population living with HIV [2]. Additionally, in 2019, sub-Saharan Africa accounted for 88% of children and adolescents living with HIV worldwide [3].

Although interventions to prevent vertical transmission have been implemented, cases of perinatal HIV transmission continue to occur [4-8]. The United Nations (UN) in the year 2002 adopted a four-pronged approach to vertical transmission prevention: 1) primary prevention of HIV infection among women of childbearing age; 2) preventing unintended pregnancies among women living with HIV; 3) preventing HIV transmission from a woman living with HIV to her infant; and 4) treatment, care, and support to women living with HIV, their children, and families [9].

However, vertical transmission prevention programs have historically focused more attention on identifying HIV infection among pregnant women, rather than on preventing HIV in women of reproductive age and preventing unintended pregnancies in women living with HIV [9]. Additionally, in most African settings, the emphasis of family planning has been primarily focused on the female partner. Little attention is paid to the dominant role the male partner plays in decision-making on family size. Therefore, focusing attention on the male partners´ involvement in family planning and contraceptive use can significantly prevent vertical transmission of HIV through the prevention of unintended pregnancies.

Among HIV-positive women, antiretroviral therapy (ART), as well as adequate knowledge regarding transmission and prevention of HIV, helps partners have an HIV-uninfected child [10,11]. Some studies have shown that in sub-Saharan Africa, HIV-positive women are more likely to have unintended pregnancies compared to HIV-negative women [12,13]. Therefore, the family planning and contraception choices of adult PLWHIV play a significant role in the prevention of vertical transmission of HIV.

No study has been conducted on the use of family planning methods among adult PLWHIV in this regional hospital with this large patient group. This study, therefore, aimed to determine the utilization of family planning methods among adult PLWHIV, to identify factors associated with the use of family planning methods, and to determine the fertility desires of the adult PLWHIV.

## Methods

**Study design and setting:** the study was a cross-sectional study conducted at the HIV clinic of the Kumasi South Hospital, Kumasi, Ghana, from October 2022 to March 2023. The hospital is the regional hospital of the Ashanti region of Ghana. The Ashanti region is located in the middle part of Ghana and is the second most populated region in Ghana, with over 5 million residents [14]. There are about 1,600 health facilities in the region, and the Kumasi South Hospital is the only regional hospital serving a large proportion of the population of the region [15]. In 2022, the Ghana Aids Commission estimates showed that the Ashanti region had the fourth highest prevalence of HIV cases in Ghana, with over 72,000 PLWHIV in the Ashanti region. The Kumasi South Hospital runs the second-largest HIV clinic in the Ashanti region. The HIV clinic runs for 5 days a week (Monday - Friday). At the time of this study, there were over 4000 PLWHIV enrolled at the HIV clinic at the Kumasi South Hospital receiving treatment.

**Study population:** the scope of this study was limited to adults presenting for care at the HIV clinic from October 2022 to March 2023. Adults presenting for care at the HIV clinic were screened for eligibility. Inclusion criteria included HIV-positive men of all ages and HIV-positive women of reproductive age (18-45 years) who consented to be part of the study. Excluded from the study were all HIV-positive women outside the age groups 18 - 45 years presenting for care at the HIV clinic at Kumasi South Hospital and all HIV-positive men of all ages and HIV-positive women of reproductive age (18 - 45 years) who did not give consent to be part of the study. The proportion of patients with HIV who used contraception was about (0.42) 42% from a study in Ghana [16]. Using this number as the national modern contraceptive prevalence and considering a confidence interval of 95% = 0.95, a Z-score of 1.96 and a level of precision of 0.05, the Cochran formula [17] was used to calculate the sample size:


$$n=\frac{z^{2}p\left(1-p\right)}{d^{2}}$$

Where n is the sample size, z is the level of confidence. Using 95% confidence interval, the Z-score is 1.96; p is the prevalence of contraception use, which is 0.42 in this case (42%); d is the margin of error or level of precision, which is 0.05 or 5%.

The minimal sample size obtained for the study was 374. A non-response rate of 15% was added. That is 15/100 x 374 = 56.1. Thus, the adjusted minimum sample size became 374 + 56 = 430. Even though we obtained this number, to further increase the power of finding a significant association, we increased the sample size to about 600 (602).

**Data collection:** adults presenting for care at the HIV clinic from October 2022 to March 2023 were screened for eligibility when they presented to the clinic for the first time. Eligible participants were randomly sampled and interviewed (n=602) after the study had been explained and participants consented to be part of the study. Informed written consent was obtained through the administration of a specially designed consent form. A structured data collection tool (questionnaire) was pre-tested and approved by the ethics committee. An interviewer administered the questionnaire. Variables assessed included sociodemographic characteristics, consistent utilization of family planning methods, number of children desired, number of children they have, HIV status of participants´ children, and barriers to the use of family planning methods ascertained two months prior to the interview day. No patient-identifiable information was collected with the questionnaire to ensure confidentiality. Identification numbers generated at the time of data collection were to remove data duplication and not for participant identification.

**Definitions:** consistent use of contraception was defined as the use of contraceptive methods during every sexual intercourse.

**Statistical analysis:** data were transferred to a Microsoft Excel sheet and imported into StataMP 17. Data were then analyzed using descriptive summary analyses. Using univariable and multivariable logistic regression, factors associated with consistent contraceptive use were identified at a statistical significance of p≤0.05.

**Ethical considerations:** permission for the conduct of the study was granted by the regional hospital´s administration. Ethical approval for the conduct and subsequent publication of the study was obtained from the Institutional Review Board for Research and Development (IRB/R&D) of Komfo Anokye Teaching Hospital (KATH), Ghana, with reference number KATH IRB/AP/102/22. The data obtained were completely anonymized using code numbers. The hard copies were securely kept under lock and key, and the electronic data was stored in a password-protected computer. The data were accessible only to the investigators. No patient-identifiable information was documented. Identification numbers generated at the time of data collection were solely to remove data duplication and not for participant identification. There was no envisaged risk to the participants.

## Results

**General characteristics of the study population:** of the total of 602 study participants, the majority (n=363) were 40-49 years old, 77.7% were females, more than two-thirds (69.8%) were Christians, most (79.7%) had a primary level of education, 88.6% were self-employed, and 76.8% were married (Table 1). Table 2 shows that the majority of the study participants have 1-3 children (83%), with 11% having 4-5 children. Most of the participants (76%) expressed the desire to have more children (4-6 children). Almost 85% of the study participants had 1-3 children alive. Almost all participants were aware of the serostatus of their children, with over 90% of them having seronegative children and 5% of participants having seropositive children.

**Table 1:** demographics of study participants from the HIV clinic of the Kumasi South Hospital, Ashanti Region, Ghana

Demographics	n	%
**Age**		
18-30	62	10.3
31-39	177	29.4
40-49	363	60.3
**Sex**		
Females	468	77.7
Male	134	22.3
**Religion**		
Christians	420	69.8
Muslims	178	29.6
Others	4	0.6
**Level of education**		
None	16	2.7
Primary	480	79.7
Secondary	96	15.9
Tertiary	6	1.0
Postgraduate	4	0.7
**Occupation**		
Unemployed	34	5.6
Self-employed	533	88.6
Private	5	0.8
Public	30	5.0
**Marital status**		
Single	52	8.6
Married	462	76.8
Separated	27	4.5
Divorced	55	9.1
Widowed	6	1.0

**Table 2:** number of children, desired number of children, number of children alive, and serostatus of children for study participants at the HIV clinic of the Kumasi South Hospital

Number of children	n	%
0	28	4.6
1-3	499	82.9
4-5	68	11.3
>5	7	1.2
**Number of children desired**		
1-3	86	14.3
4-6	455	75.6
>6	61	10.1
**Number of children alive**		
0	26	4.3
1-3	511	84.9
4-6	62	10.3
>6	3	0.5
**Any seropositive children**		
Yes	30	5.0
No	546	90.7
Don't know	26	4.3

**Figure 1:**
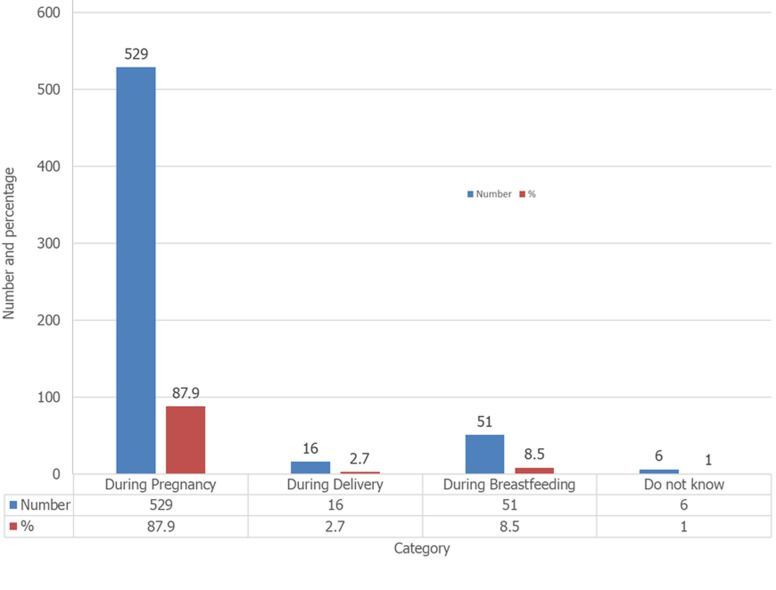
study participants' knowledge on vertical transmission of HIV

**Human immunodeficiency virus infection and knowledge of vertical transmission and partner´s serostatus:** as shown in Table 3, there was an increased survival of PLWHIV in this study population, with over 77% of study participants diagnosed with HIV infection 4-6 years ago. Over 99% of participants were on antiretroviral therapy. The majority of the partners of study participants had primary (56.2%) or secondary (36.5%) levels of education and were self-employed (79.0%). Almost 90% of study participants knew the serostatus of the partners, 87.8% of participants with seronegative partners, and 1.3% of participants with seropositive partners. There were comparable rates of knowledge of partners´ serostatus among both male (89.6%) and female (89.0%) study participants. Figure 1 also shows that study participants showed a high level of knowledge on the vertical transmission of HIV. Over 99% of participants said they had received education on HIV vertical transmission. Almost 88% of participants indicated that vertical transmission of HIV occurs during pregnancy.

**Table 3:** duration of HIV infection, treatment of HIV, knowledge of partner’s serostatus, partner’s level of education, and occupation of study participants

Duration of HIV infection (years)	n	%
<1	3	0.5
1-3	46	7.6
4-6	465	77.3
7-9	73	12.1
>10	15	2.5
**Study participants on ARVs**		
Yes	598	99.3
No	4	0.7
**Serostatus of partner**		
Positive	8	1.3
Negative	525	87.2
Don't know	69	11.5
**Partner's educational status**		
None	26	4.3
Primary	338	56.1
Secondary	219	36.5
Tertiary	17	2.8
Postgraduate	2	0.3
**Partner's occupation**		
Unemployed	13	2.2
Self	474	78.7
Private	8	1.3
Public	107	17.8

ARVs: antiretrovirals

**Use of family planning methods:** almost all study participants (98.9%) had received family planning counseling. Of these, 93.9% received the counseling after diagnosis of the HIV infection. Over 97% of participants currently use any family planning method consistently. Consistent use of family planning methods was comparable between female (96.8%) and male (98.5%) study participants. The barrier contraceptive method was the most widely used method among study participants (98.6%). Almost 97% of study participants had been offered family planning assistance before, with 95.3% of participants receiving the assistance after HIV diagnosis. The majority of study participants had ever used a family planning contraceptive method, with 98.6% of participants using a barrier contraceptive method. The barrier contraceptive method was the most familiar method known to almost all participants (98.8%). However, only 10.6%, 7.3%, and 3.8% of participants were familiar with natural methods, hormonal contraceptives, and intrauterine devices, respectively. These results are depicted in Table 4.

**Table 4:** use of family planning methods among study participants

Current consistent use of family planning methods	n	%
Yes	585	97.2
no	17	2.8
**If yes, which method**		
Barrier contraceptive	572	98.6
Hormonal	7	1.2
Intrauterine	1	0.2
**Ever used a family planning method?**		
Yes	590	98
No	12	2
**Family planning method ever used**		
Natural method	16	2.7
Barrier contraception	582	98.6
Hormonal contraception	34	5.8
Intrauterine contraceptive device	1	0.2
Surgical contraception	1	0.2
**Receipt of any family planning counselling**		
Yes	595	98.8
No	7	1.2
**If yes, was it before or after HIV diagnosis**		
After	565	93.9
Before	30	6.1
**Ever offered any family planning assistance?**		
Yes	583	96.8
No	19	3.2
**If yes, was it before or after HIV diagnosis?**		
After	564	95.3
Before	28	4.7
**Which family planning methods are you familiar with?**		
Natural methods	64	10.6
Barrier contraceptives (condom, diaphragm, etc.)	594	98.8
Hormonal contraceptives (injectables, COCPs, subdermal)	44	7.3
Intrauterine device (hormonal or copper IUD)	23	3.8
Surgical	16	2.7
None	4	0.7

COCPs: contraceptive pills for perimenopause; IUD: intrauterine devices

**Factors associated with consistent use of contraceptives:** marital status, desired number of children, duration of HIV infection, partner´s serostatus, and receipt of family planning assistance were significantly associated with the increased odds of consistent use of family planning methods. The odds of consistent family planning utilization among married study participants were increased compared to those of unmarried patients (aOR-17.00, 95% CI: 3.50-82.40; P<0.001). The odds of consistent use of family planning methods among people living with HIV infection for 4 years or more were 9.34 times that of people living with HIV infection for 3 years or less (aOR- 9.34 95% CI: 2.41-36.15; P=0.001). Patients who received family planning assistance had an increased odds of consistent family planning method use. Partner´s HIV status also influences the consistent use of contraceptives. Study participants whose partners have positive serostatus also had lower odds of consistent family planning method use (aOR- 0.07, CI: 0.01- 0.74; P=0.028) compared to participants with partners with negative serostatus, as shown in Table 5. Study participants who desire to have 4 or more children had decreased odds of consistently using contraceptives relative to participants who desire to have 3 or fewer children; however, this was found to be insignificant (aOR- 0.12, 95% CI: 0.10-1.03; P=0.053).

**Table 5:** unadjusted and adjusted odds ratios of factors associated with consistent family planning use among study participants

Variables	Unadjusted OR (95% CI)	p-value	Adjusted OR (95% CI)	p-value
**Marital status**				
Not married	Reference		Reference	
Married	27.60 (6.20-122.30)	<0.001*	17.00 (3.50-82.40)	<0.001*
**Duration of HIV infection**				
≤3 years	Reference		Reference	
≥4 years	9.05 (3.27-24.99)	<0.001*	9.34 (2.41-36.15)	0.001*
**Family planning assistance**				
Not received	Reference		Reference	
Received	28.89 (8.31-100.37)	<0.001*	19.54(3.36-113.57)	0.001*
**Partner’s serostatus**				
Negative	Reference		Reference	
Positive	0.08 (0.01-0.42)	0.003*	0.07 (0.01-0.74)	0.028*
**Desired number of children**				
1-3 children	Reference		Reference	
≥4 children	0.80 (0.18-3.54)	0.764	0.12 (0.1-1.03)	0.053

* P-value<0.05

## Discussion

This study aimed to determine the utilization of family planning methods among adult PLWHIV, to identify factors associated with the use of family planning methods, and the fertility desires of the adult PLWHIV. This study has shown a high level (over 97%) of consistent use of family planning methods among PLWHIV. This could be attributed to the family planning counseling that patients receive after HIV diagnosis, as evidenced by the over 94% admitting to having received such counseling. Comparative studies have also demonstrated a high level of consistent use of family planning methods in PLWHIV, with values such as 73.1% and 74.5% [18,19]. A comparable study at the Komfo Anokye Teaching Hospital in Kumasi also demonstrated high utilization of family planning methods and was strongly associated with family planning counseling upon HIV diagnosis and partner knowledge of HIV status [16]. Some studies have, however, reported an average to very low utilization of family planning methods with values such as 44.3%, 46%, 51.2% and 16.8% [20-23].

The high utilization of family planning may therefore be rooted in the programmatic family planning counseling for all newly diagnosed PLWHIV in Ghana. This study showed that the barrier contraceptive method was the most popular form of contraceptive among study participants. This may be attributed to barrier contraceptive methods, such as condoms, being easily accessible compared to other methods, including intrauterine contraceptive devices. Also, patients are aware of the crucial role of the use of barrier contraceptive methods in reducing the transmission of HIV infection through family planning counselling programs, which may make this method the most preferable choice of contraception. This, however, pushes bias towards only the use of condoms due to the emphasis on prevention of HIV transmission. Also, there is a high risk of unwanted pregnancy associated with the use of only barrier methods. Barrier contraceptive methods were associated with 18 or more unintended pregnancies out of every 100 women compared to less than 1 unintended pregnancy per 100 women in women who use long-term reversible contraceptive methods such as an implant or intrauterine contraceptive devices [24]. To reduce unintended pregnancies as well as HIV infection transmission, people living with HIV should be educated on using a combination of long-term reversible contraceptives and barrier methods. Long-term reversible contraceptive methods should be made readily available for PLWHIV.

In addition to the increased level of family planning use, the study participants showed a high rate of treatment and knowledge of the serostatus of partners and their children, with comparable rates between male and female study participants. There is the perception that there are low levels of knowledge of partners' HIV serostatus, especially among women, due to poverty, with associated low bargaining power. A study done among pregnant women at the Kumasi South Hospital corroborated this perception, demonstrating a low knowledge of partner HIV serostatus [25]. This study, however, demonstrated a rather high rate of knowledge of the serostatus of partners. This finding has also been seen in a study in Uganda, which demonstrated very high knowledge of the serostatus of partners, especially among married couples [26]. Partner and religious opposition to contraceptive use and fear of side effects of contraceptives were the key concerns expressed by study participants. These factors have been demonstrated by other studies to influence the use of family planning methods among women living with HIV [27-30]. The majority of the study participants had fertility desires. Other studies in Ghana have shown high fertility desires [31]. Fertility desires are also strong in PLWHIV in other African countries [32,33].

Factors associated with consistent use of contraceptive methods included receipt of family planning assistance, prolonged duration of HIV infection (≥4 years), and being married. These factors have been shown in other studies to be associated with family planning use [28,34]. The provision of family planning assistance provides patients with the necessary education and empowerment to make an informed decision on contraception use. Married PLWHIV are more likely to have the social support needed to make an informed decision on the consistent use of contraception. Healthcare providers must identify single patients with a short duration of infection and provide targeted family planning assistance to improve the consistent use of family planning methods. Also, various sources of social support from healthcare providers and family members must be explored to ensure the family planning needs of PLWHIV are met to reduce unintended pregnancies.

Factors such as having a seropositive partner and desiring to have 4 or more children were associated with less consistent use of contraception, as shown in other studies [35,36]. Dual HIV 1 and 2 coinfections, which are common in West Africa, are a risk among PLWHIV with seropositive sexual partners, increasing mortality and morbidity in this group [37]. Persons Living with HIV must be educated on the potential risk of dual HIV-1 and HIV-2 and the need to use a combination of barrier and long-term reversible contraceptive methods, irrespective of the serostatus of their partners, to reduce HIV transmission or dual HIV infection and unintended pregnancies. PLWHIV with a desire to have more children should be supported, educated, and provided with effective contraceptive options to ensure adequate spacing whilst preventing unintended pregnancies.

This study has certain limitations. Precategorization of continuous variables limited the measurement of central tendencies and deviations. There could be biases in collecting data on the primary outcome of interest of this study. The use of contraceptive methods is based on self-reported data. There is, therefore, the possibility of recall bias affecting participants' recollection of their use of family planning methods. The study was conducted at an HIV clinic in a regional hospital with more resources compared to other HIV clinics in the country. Generalization of the results must therefore be done with caution.

## Conclusion

This study into family planning utilization among people living with HIV showed that barrier contraception is the most widely used. Consistent use of contraception among PLWHIV was influenced by marital status, HIV infection duration of 4 or more years, and receipt of family planning assistance. Having a seropositive sexual partner was associated with less consistent use of contraception. Even though the barrier method is most effective at reducing HIV transmission, it is less effective at reducing unintended pregnancies when compared to long-term reversible contraceptives. To reduce unintended pregnancies and the rate of HIV transmission, as described by the UN four-prong approach, education on contraceptive use among PLWHIV should be pivoted towards dual contraception. PLWHIV with characteristics associated with less consistent use of contraception must be identified, educated, and supported to improve contraception use and reduce unintended pregnancy.

### What is known about this topic

- There is a high unmet need for family planning in PLWHIV in Ghana;
- Partner knowledge of HIV status and previous contraceptive use are strong predictors of contraceptive use among PLWHIV.

### What this study adds

- Barrier contraception is the most widely used among PLWHIV;
- Consistent use of contraception among PLWHIV was influenced by marital status, HIV infection duration of 4 or more years, and receipt of family planning assistance;
- Having a seropositive sexual partner was associated with less consistent use of contraception.
